# Protein and Sport: Alternative Sources and Strategies for Bioactive and Sustainable Sports Nutrition

**DOI:** 10.3389/fnut.2022.926043

**Published:** 2022-06-17

**Authors:** Manuel I. López-Martínez, Marta Miguel, Marta Garcés-Rimón

**Affiliations:** ^1^Departamento de Bioactividad y Análisis de Alimenos, Instituto de Investigación en Ciencias de la Alimentación (CIAL, Consejo Superior de Investigaciones Científicas-Universidad Autónoma de Madrid), Madrid, Spain; ^2^Grupo de Investigación en Biotecnología Alimentaria, Universidad Francisco de Vitoria, Madrid, Spain

**Keywords:** sports nutrition, protein hydrolysates, alternative protein sources, bioactive peptides, amino acids

## Abstract

Nutrition and sport play an important role in achieving a healthy lifestyle. In addition to the intake of nutrients derived from the normal diet, some sport disciplines require the consumption of supplements that contribute positively to improved athletic performance. Protein intake is important for many aspects related to health, and current evidence suggests that some athletes require increased amounts of this nutrient. On the other hand, society's demand for more environmentally friendly products, focus on the search for alternative food sources more sustainable. This review aims to summarize the latest research on novel strategies and sources for greener and functional supplementation in sport nutrition. Alternative protein sources such as insects, plants or mycoproteins have proven to be an interesting substrate due to their high added value in terms of bioactivity and sustainability. Protein hydrolysis has proven to be a very useful technology to revalue by-products, such as collagen, by producing bioactive peptides beneficial on athletes performance and sport-related complications. In addition, it has been observed that certain amino acids from plant sources, as citrulline or theanine, can have an ergogenic effect for this target population. Finally, the future perspectives of protein supplementation in sports nutrition are discussed. In summary, protein supplementation in sports nutrition is a very promising field of research, whose future perspective lies with the search for alternatives with greater bioactive potential and more sustainable than conventional sources.

## Introduction

Nutrition and sports play an important role in maintaining a healthy lifestyle (1, 2). Sports nutrition is a multidisciplinary science which includes fields such as anatomy, biomechanics, biochemistry, physiology and nutrition (3). The number of studies that found a relationship between sports and diet has increased over the last 20 years, and nowadays, there is no doubt about the essential role that nutrition plays in sport. In addition, nutrition should be adapted according to the characteristics of the sport, and the importance of the personalized nutrition in sport performance is being widely studied (4). Moreover, sports nutrition is one of the fastest growing segments of the functional foods and dietary supplement markets.

In addition to the intake of nutrients derived from the normal diet, some sport disciplines require the consumption of supplements that contribute positively to improve sports performance. These supplements, known as ergogenic aids, are defined as substances used to improve endurance, total fitness level and sports performance (5). Some of them, as creatine, caffeine, bicarbonate, protein and amino acids, have been used for different purposes: to increase energy intake, to maintain strength, to recover muscular mass or to prevent nutritional deficiencies, among others (6–9). Two of the most marketed supplements in sports nutrition are proteins and amino acids. In fact, 80% of sports nutrition sales come from protein-based products, mainly in bars and powder formats (10), although the development of functional protein beverages is playing an important role in recent years (11). On the other hand, the current demand for more environmentally friendly products has increased research into more sustainable alternative sources of protein (10).

The purpose of this review was to summarize the latest research regarding novel strategies for protein supplementation in sports nutrition, focus on alternative protein sources (insects, plant-based and mycoproteins), highlighting its added value provided in terms of bioactivity and sustainability. The benefits of protein hydrolysates and bioactive peptides on athlete's performance, sports-related complications and healthy aging and the ergogenic effects exerted by novel amino acids obtained from natural sources were also discussed. Furthermore, future perspectives for protein supplementation in sports nutrition are addressed.

## Proteins

Proteins are a key macronutrient to maintain a good nutrition and health status, and necessary for the proper functioning of the organism. Among these functions, proteins ensure growth in childhood, support muscle and bone metabolism, contribute to the maintenance of the nervous system and help to maintain muscle mass and physical performance in later life (12). Net protein balance (NPB) is defined as the difference between skeletal muscle protein synthesis (MPS) and muscle protein breakdown (MPB). Therefore, a significant increase in skeletal MPS (anabolism) and/or decrease in MPB (catabolism) can lead to an increase in skeletal muscle mass (13). An adequate intake of proteins in the diet is important for maintaining a good health condition, and current evidence has suggested that athletes of some sport disciplines need to consume greater amounts of protein. Generally, athletes require higher amounts of high-quality protein in their diet (1.2–2.0 g/kg/day) compare to non-athletic adults (0.8–1.0 g/kg/day) and, in particular, rich in branched-chain amino acids (BCAAs), to stimulate MPS and promote a positive protein balance (8, 14). Leucine, Valine, and Isoleucine constitute the group of branched-chain amino acids (BCAAs). Around 33% of the skeletal muscle is composed of a combination of these three essential amino acids (15). Unlike other essential amino acids, BCAAs are metabolized directly in the muscle tissue and they are the first energy source used during exercise (16). In addition, these amino acids, specially leucine, promote insulin secretion, reduce central fatigue, decrease muscle soreness and mainly increase MPS by the activation of the mammalian target of rapamycin (mTOR) pathway (17).

In nutrition, the term “protein quality” focuses on the relative composition of amino acids present in a protein source compared to a protein pattern that is considered to represent the highest quality protein (18). The Food and Agricultural Organization of the United Nations/World Health Organization (FAO/WHO) and FDA first proposed a protein quality evaluation method in 1991, the Protein Digestibility-Corrected Amino Acid Score (PDCAAS), which became the official standard in 1993. Although they included the relevance of the digestibility in the score, it also depends on numerous factors, including the amino acid absorption kinetics and the capacity of the particular dietary protein to stimulate protein synthesis in the organism (19). Consequently, in 2013, FAO proposed a new evaluation method, the Digestible Indispensable Amino Acid Score (DIAAS). This score is a more accurate rating of protein quality. Furthermore, DIAAS samples come from the ileum, while PDCAAS samples come from feces. If there are no amino acids in the feces samples, the PDCAAS method assumes digestion. But the DIAAS samples from the ileum are a more accurate representation of amino acid digestion and absorption (20).

In the last decades, the most commonly used food protein sources in sports nutrition have been milk, egg and meat (10). All of them are considered foods with high-quality protein. Moreover, ovalbumin, a protein from egg white, has been used as standard for comparing other dietary proteins (21) due to its complete amino acid profile and its excellent digestibility, followed by whey protein (15). Worldwide, whey protein is the most consumed protein as a supplement in sports nutrition. This protein, obtained as a by-product in the production of cheese and butter, is very interesting both, economically, since it is considered a cheap by-product, and nutritionally, as a high-quality protein with a notable content in essential amino acids and BCAAs (22). There are numerous studies that prove its efficacy in sport performance, such as muscle recovery (23, 24), increment of strength or changes in body composition (25). In addition, whey proteins have shown several biological properties such as antioxidant, anticancer, antidiabetic, anti-obesity or cardioprotective activities, among others (26). These multiple benefits support whey protein as an effective sports supplement. However, the current growth of the world's population, combined with increasingly limiteresources, has resulted in the need for alternative protein sources to meet global protein requirements, including sports nutrition (27).

### Insect Proteins

Insects, belonging to the largest class of phylum *Arthropoda*, are small animals with segmented bodies, jointed legs, and exoskeletons. This animal group includes more than a million of species and represents more than half of all known living organisms (28). Insects are institutionally accepted as food in many regions and historically consumed (29) providing an interesting nutritional value for humans (30). Worldwide, the most consumed species of insects have been studied to determine their nutritional value and organoleptic properties. In fact, the European Regulation 2015/2283 on new foods, entered into force in January 2018, and allowed the sale of insects at a commercial level for their use and consumption in the European Union (31).

In last decade, insects have been considered one of the most environmentally friendly sources of animal protein, as its carbon and water footprint is very low (32). The insects that are most commonly used as food are species belonging to the order *Coleoptera (beetles), Lepidoptera, Hymenoptera and Orthoptera* (32). In general, these insects contain large amounts of chitin, an insoluble polysaccharide composed of N-acetylglucosamine units, belonging to its exoskeleton. They also provide high levels of vitamins and minerals as well as an excellent production efficiency compared to other conventional food sources (33). Insects have a high protein content (40–60%) comparable to traditional animal protein sources as egg, milk o meat (34). In addition, they have a good amino acid profile, with an average in most of the EAAs and BCAAs higher than the recommended by the World Health Organization (WHO) (35, 36). Moreover, the average leucine content, one of the most important BCAA due to its role as a stimulator of muscle protein synthesis (37), is higher in *Coleoptera, Hymenoptera, Isoptera* and *Orthoptera*, than soy (63 mg leucine/g soy protein to 75–80 mg leucine/g insect protein) and comparable to skim milk powder (77 mg/g protein) and micellar casein (82 mg/g protein) (34, 36). Furthermore, proteins from insects have relatively high digestibility, with values among 54% for *Tenebrio molitor (Coleptera)* (38) to 90.5% for grasshopper (*Orthoptera*) (39). However, this parameter depends on their composition and how insects are processed. In this regard, presence of chitin can have a negative effect on digestibility (39). Therefore, the final product should reduce the chitin levels as much as possible, in order to achieve an optimal protein digestibility (34).

Although there are not many studies that relate insect protein consumption and sports, some of them have shown promising results. Vangsoe et al. (40), conducted a randomized control trial in which they compared the effect of 25 g of whey, soy or insect protein isolates on the postprandial increase of amino acids in blood of athletes. They observed that insect protein isolate intake raised blood EAAs and BCAAs concentrations similar to soy protein isolates, suggesting that they could be used as alternative source for MPS (40). These authors also reported in other study that insect protein isolate supplementation (0.8 g/kg) exerted an increase in fat free mass and muscle strength after 8 weeks of resistance training. However, these differences were not significant with the control, suggesting that the doses of insect protein isolate used should be higher (41). More recently, Hermans et al. (42), conducted a double-blind randomized controlled trial in which they compared the ergogenic effects of 30 g of lesser mealworm protein isolates with 30 g of milk protein isolates. Lesser mealworm protein consumption increased the post-prandial muscle synthesis rate both at rest and during exercise recovery, in a similar way to the results observed for milk protein. These results suggest that lesser mealworm protein can exert an ergogenic effect similar to that of a traditional high biological value protein, such as milk protein (42).

Overall, insect protein isolates could become an alternative ingredient for post-exercise recovery nutrition in sports products, since they are considered a high-quality protein source, due to their amino acid profile and digestibility. However, it would be convenient to study the environmental impact of its isolates, since chitin is produced as a by-product, thus being a non-zero waste product.

### Plant-Based Proteins

The evidence on the benefits of some animal proteins in sport performance has already been proven. Nevertheless, the demand for clean-label, non-allergenic and plant-based products is increasing. Sustainability, animal welfare and ethics-related concerns are driving the demand for new alternatives to animal proteins (43, 44). A well-designed vegan diet could provide enough energy and an adequate range of carbohydrate, fat and protein intake to support performance (45). In fact, it seems that some plant-based proteins could be as effective as animal-based proteins for muscle maintenance, providing all the essential amino acids (46).

In recent years, several human trials have been conducted using plant proteins as sport supplements with promising results. These studies are compiled in Table 1. In general, vegetable protein supplementation provides similar ergogenic effects to those generated with animal protein, as an increase in strength (50), an improvement in MPS (48, 52) or a reduction in body fat mass (51). However, the anabolic response in most of them, with the exception of soybean (53), was lower than the reported with whey protein. This fact is mainly due to the amino acid composition of vegetable proteins. Plant-based proteins are characterized by a lower bioavailability and a lower content of EAA and BCAA than traditional animal proteins, as whey or egg proteins (27). Moreover, it has been proven that some vegetable proteins mainly from legumes, as pea or soy protein, do contain the WHO recommended percentage of EAA (35). The physical and chemical composition of the food matrix and the structure of its proteins make vegetables a source of protein more resistant to proteolysis, which leads to a reduction in digestibility. Therefore, the isolation of these proteins to include in other food matrix or the use of different proccesing methods or culinary preparations could help to increase its digestibility (54). In fact, food products based on these proteins, such as tofu or soy milk, are currently one of the most marketed products as substitutes for animal-based foods and it has recently been observed that its digestibility is higher than that starting raw material (55). It is important to note that in addition to their deficiency in certain amino acids, vegetable proteins can be reduced in quality due to the presence of anti-nutrients. Anti-nutrients are substances with no nutritional value that have several effects on the body. Among them, those that most affect protein digestibility and bioavailability are the protease inhibitors, as the Kunitz or Bowman Birk. Both compounds, present in legumes such as soybeans, reduce the effect of trypsin in the organism, causing a decrease in the digestion consumed proteins (56). However, there are certain simple strategies, such as soaking or heat treatment that significantly reduce these anti-nutrients. Moreover, it has recently even been observed that techniques well suitable to the production of sports supplements, such as wet extrusion, are also very effective in eliminating these anti-nutritional factors (57).

**Table 1:** Summary of plant-based proteins clinical studies in sports nutrition.

	**Type of trial**	**N°** **participants**	**Source**	**Samples**	**Protein dose and timing**	**Trial period**	**Remarkable effects**	**References**
1	Double-blind, randomized, placebo-controlled trial	40 trained males	Soybean	Target: Soybean protein Placebo: Aspartame	50 g/day After training in two times	4 weeks	↓ Muscle damage compared to placebo (CP) ↓Oxidative stress increment CP ↑ Muscle recovery CP	(47)
2	Double blind, randomized, single dose, parallel-group trial	60 healthy old men	Wheat	Target: Wheat protein Control 1: Casein Control 2: Whey protein	35 g/day (controls) 60 g/day (Target) After the trial	1 day	↑Myofibrillar protein synthesis No significant differences with the positive control (NSD)	(48)
3	Double-blind, randomized, placebo-controlled trial	16 healthy untrained men	Oat	Target: Oat protein Placebo: Maltodextrin	25 g/day Before training Consecutive 4 days after training	19 days	↓ Muscle soreness CP ↓Inflammation markers CP ↑markers Cecovery CP	(49)
4	Double-blind, randomized, comparative trial	15 athletes	Pea	Target: Pea protein Control: Whey protein	24 g/day Before and after training	8 weeks	↑ Muscle strength NSD	(50)
5	Double-blind, randomized, prospective, two-group parallel-arm trial	48 untrained people	Soybean	Target: Soybean protein Control: Whey protein	26 g/day (Target) 19 g/day (Control) After training	12 weeks	↑Muscle strength ↓Fat body mass NSD	(51)
6	Double blind, randomized, single dose, parallel-group trial	36 healthy untrained men	Wheat	Target: Wheat protein Control: Milk protein	30 g/day After the trial	1 day	↑Myofibrillar protein synthesis NSD	(52)

Although protein quality is generally lower in plants than in animals, there are other reasons to choose them as sports supplements. In this sense, several studies affirm that diets rich in vegetables contribute to reduce oxidative stress and inflammation (54, 58). Oxidative stress is the consequence of an imbalance between the production of reactive oxygen species (ROS) and the body's antioxidant defense. This adverse condition can cause serious tissue and cellular damages and it is related to many disorders such as inflammation, cardiovascular diseases, and cancer (59). The relationship between exercise and oxidative stress is extremely complex. Since regular moderate training seems to be beneficial for health, it has been described that acute exercise increases oxidative stress and ROS production *via* lipid peroxidation, superoxide anion generation and incrementation in oxidized/reduced glutathione (GSSG/GSH) ratio (59–61). The increment of ROS can generate an overproduction of proinflammatory cytokines, which can lead to muscle fragility by reducing myofibrillar protein synthesis and increasing protein catabolism, leading into a negative impact on sport performance. In addition, this effect can be cyclical, generating a chronic problem (62). In this context, oral antioxidant supplementation could be a suitable non-invasive tool to prevent or reduce oxidative stress during training (63), and plant protein sources could be used for this purpose (47, 49, 60, 64, 65). In this regard, polyphenols, such as avenanthramides (66), carob phenols (67) or soy isoflavones (65) including in vegetable foods, could promote the reduction of inflammation and oxidative stress. They could be beneficial for athletes to maintain their health status after sports performance.

Besides polyphenols, proteins from plants also contain several bioactive compounds that could contribute positively to improve the performance in sportspeople. In this sense, β-conglycinin, a storage protein from soybean, improved glucose uptake in skeletal muscle (68). In addition, β-glucans from oats, a soluble dietary fiber, could contribute to the maintenance of plasma glucose levels (69).

Taken together, research into the use of plant protein sources as sports supplements could be very interesting, as they could become an ergogenic, sustainable and value-added products for athletes, especially for those who exercise intensively, due to its contribution to reducing oxidative stress.

### Mycoproteins

Other protein sources could also offer several benefits as sports supplements are mycoproteins. Mycoproteins are a sustainable food source derived from different species of fungi, mainly *Fusarum venenatum* (70). In relation to their nutritional value, mycoproteins are rich in dietary fiber (β-glucans and chitin), present high-quality proteins (~45% of total mass) and a biological value similar to traditional meats (84 vs. 80%). Moreover, mycoproteins are rich in essential amino acids (41% of total protein) and possess a high digestibility (0.99), similar to animal protein sources as milk (71, 72).

In the last decade, mycoproteins have emerged as an environmentally friendly and cheap source, since it can be synthetised with agronomical by-products and its carbon footprint is, at least, 10 times lower than traditional meats (73). Furthermore, mycoproteins appeared not to be potentially allergenic (74) and they could exert benefits related to sports nutrition and cardiometabolic health, such as insulinemia and glycemia attenuation and muscle adaptation (72).

In recent years, several authors analyzed the possible ergogenic effect of mycoproteins in sports nutrition. In this sense, Dunlop et al. (75), compared the effect between 20 g of whole food mycoproteins and 20 g of milk protein concentrate intake on acute postprandial hyper aminoacidemia and hyperinsulinemia, to analyse their anabolic effect, which is directly related to these two parameters. These authors concluded that mycoprotein ingestion resulted in a slower but more sustained hyperinsulinemia and hyper aminoacidemia compared to milk protein ingestion. Moreover, it means that mycoproteins could be a useful dietary protein source with an noticeable anabolic effect in muscles (75). More recently, Monteyne et al. (76), performed a randomized controlled trial, in which they compared the ergogenic effect that a simple 70 g bolus of whole food mycoproteins or leucine-enriched milk protein could have on athlete's MPS. The results showed that mycoproteins exerted a higher postprandial induction on protein synthesis (0.040 ± 0.006 %/h) than leucine enriched milk proteins (0.018 ± 0.005 %/h), regardless of their consumption before or after training. This research indicates that this source of proteins could have a superior anabolic effect than traditional proteins used in sport supplements (76). These authors also investigated whether a simple bolus with a lower whole food mycoprotein content (35 g) enriched with BCAAs could exert the same effect as a simple 70 g bolus of whole food mycoprotein. They found that a higher dose of mycoproteins (70 g) stimulated muscle synthesis more than a lower dose (35 g) supplemented with BCAAs (77). Finally, these authors conducted a study in which they aimed to determine whether a vegan diet supplemented with mycoproteins could maintain MPS in a similar way to an omnivorous diet. Their results showed that a vegan diet high in mycoproteins contributed to maintaining daily rates of muscle protein synthesis rates both at rest and after exercise, equivalent to a high-protein omnivorous diet in healthy older adults (78).

In summary, mycoproteins have proven to be a very interesting protein source for sports nutrition, due to their remarkable anabolic effect on MPS, equivalent to that of traditional sources such as milk. In addition, they are very sustainable, since in most studies they have demonstrated their effect without being isolated, which makes them an environmentally friendly protein source that does not generate waste, since the whole food is consumed.

## Hydrolysates and Peptides

Protein hydrolysates are a complex mixture of peptides, mainly di- and tripeptides, whose production is based on the cleavage of the structure of protein sources by thermal, acid, or enzymatic treatment. Their composition depends on several factors and can be defined by the degree of hydrolysis, which is the fraction of peptide bonds that have been cleaved in the native protein (79). It is generally accepted that protein hydrolysates containing mostly di- and tripeptides are absorbed faster than intact proteins (80).

In sports nutrition, hydrolysates could be advantageous over native proteins because they are pre-digested. Therefore, their peptides and amino acids could be available to the muscle more rapidly after ingestion. The increase of amino acids in plasma after ingestion of protein hydrolysates can result in a stronger stimulation of MPS compared to the whole protein (80).

In recent years, some studies confirm the hypothesis that hydrolysates seem to generate a faster muscle recovery effect than intact proteins (81, 82). It seems to be related to an increase in the bioavailability of amino acids, especially BCAA and EAA, as they have a positive effect on MPS (83). This increase in the bioavailability of amino acids is due to the fact that since they are pre-digested, the release of amino acids in the intestine is greater. In addition, it seems that peptides, especially di- and tripeptides, can be absorbed through specific transporters, leading to an even greater increase in blood aminoacidemia (84). Apparently, this hyper aminoacidemia also seems to be related to a possible thermogenic effect, which could have beneficial effects in athletes to reduce their fat body mass. Although further research is needed to confirm all these effects (85, 86). Other beneficial effects of protein hydrolysates on sport performance could be related to the generation of bioactive peptides. Bioactive peptides are defined as amino acid sequences that are not active in the precursor protein structure but that can exert physiological functions in the organism after their release through *in vivo* (gastrointestinal digestion) or *in vitro* hydrolysis (chemical or enzymatic). Since bioactive peptides were discovered in 1979, different biological activities have been described as antioxidant, antimicrobial, opioid, immunomodulatory, anti-inflammatory, antihypertensive, antidiabetic or hypocholesterolemic, among others (87, 88). In this sense, some studies reported that certain bioactive peptides may exert a positive effect on muscle glucose uptake and glycogen restore by activating insulin-independent AMP-activated protein kinase signaling in skeletal muscle cells (89, 90). Moreover, endogenous insulin appears to exert a positive effect on MPS due to their vasodilator effect by stimulating endothelial nitric oxide production, and increasing skeletal muscle glucose uptake (91). On the other hand, insulin has a similar structure to insulin-like growth factor-1 (IGF-1). IGF-1 is a hormone capable of exerting an anabolic effect on skeletal muscle. Therefore, insulin may simulate this effect (92). Some bioactive peptides could also increase plasma concentrations of glucose-dependent insulinotropic polypeptide, which implies an enhancement of insulin release from pancreatic beta cells (93). In fact, the greater insulinotropic effect observed after consumption of protein hydrolysates compared to intact proteins could explain their increase in MPS rate (94).

Other biological activities related to protein hydrolysates or their bioactive peptides such as antioxidant, immunomodulatory or anti-inflammatory properties could be especially useful to control sport performance and some complications derived from sport practice. Some of them are explained in detail below.

### Protein Hydrolysates in Joint Soreness

Strenuous exercise, especially in high-impact sports, can lead to a continued joint pain in athletes, and can affect negatively in their performance (95). Collagen is the most abundant animal protein and it is present in the connective tissue of animals, being generally a by-product of the meat industry (96). This protein is often used in comparative studies to observe the impact of protein quality on muscle recovery, being used as a negative control due to its low biological value (96, 97). However, the hydrolysis of this by-product has become a sustainable way to revalue it. In this sense, researchers have investigated the effect of specific collagen peptides (SCP) by their biological properties (98, 99), some of them related to joint soreness. Clark et al. (100), pioneered the analysis of the effect of SCP supplementation in sportsmen's joint soreness. A 24-week prospective, randomized, placebo-controlled, double blind study was conducted with 147 athletes. They consumed 10 g of a collagen hydrolysate or placebo. The results showed that a daily treatment of 10 g of a collagen hydrolysate was able to reduce joint discomfort, both at rest and during movement (100).

More recent studies have also noted the possible positive effects of these collagen derived peptides. Exercise combined with 15–20 g of collagen hydrolysates, preferably after training, for over a week, leads to an improvement in lean body mass, and reduces joint and muscle pain in athletes and physically active people (101–106). The mechanisms by which the collagen peptides exerted their effect have not yet been elucidated. However, it has been suggested that faster remodeling of affected tissues could be related to an increase in collagen synthesis in the connective tissues surrounding the muscle and/or to a modulation of the inflammatory response (107). The main limitation of human studies is that, it is often difficult to establish the mechanism of action because it is complicated to take muscle biopsy samples, analyse biomarkers or explore the signaling pathways in the tissues. Nevertheless, some authors have studied the effect of exercise and collagen peptide supplementation, using proteomic techniques on muscle biopsies. They found that some proteins, such as Collagen 5α1, associated to myofibrillar recovery, are upregulated, so this could explain the positive effects mentioned above (108, 109).

In summary, the consumption of collagen hydrolysates could represent an environmentally friendly strategy to reduce joint pain in high-performance athletes.

### Anti-fatigue Hydrolysates and Peptides

Fatigue is defined as a reduction in the maintenance of physical conditions such as power and strength, which impairs physical performance. The main causes of fatigue include: increased acidity in the muscle cells, depletion of energy sources such as phosphocreatine and glycogen, accumulation of ammonia and urea in the blood and tissues, oxidative stress and muscle inflammation (107, 110). Fatigue is a factor that should not be underestimated in sports nutrition. This condition, prolonged over time, can lead to the development of chronic fatigue syndrome, which not only affects athlete's performance, but his or her health may also be affected (111).

In recent years, it was observed in the literature that certain by-products from natural sources could be substrates to produce anti-fatigue peptides. These studies are summarized in Table 2. In general, most of these studies were conducted with murine models, in which the anti-fatigue effect of the peptides was assessed by a weight-loaded swimming test. The reduction in fatigue was reflected externally by the improvement in the time that mice can swim without becoming exhausted (115). In general, the studies referenced in Table 2 showed that the consumption of a certain amount of protein hydrolysates (200–600 mg/day) before training, and for a 4-weeks period, could lead to a very noticeable improvement in the time to exhaustion, being in several cases higher than 100%, and even better than traditional protein sources such as whey from milk (114).

**Table 2:** Summary of anti-fatigue peptides studies in rodent's model.

	**Source**	**N°** **subjects**	**Samples**	**Dose and timing**	**Increase of time of exhaustion** ^**b**^	**Effects**	**References**
1	Croceine Croaker	48 mice	Control:physiological saline Sample: Croceine croaker bladderhydrolysate.	Control: 0.1 mL/10 g Body Weight (BW)/day Sample: 50 mg−200mg/kg BW/day 30 min before exercise 28 days	57.9% - (16 min → 25.4 min) 107.5% - (16 → 33.4 min)	↑Antioxidant enzymes ↓BUN,BLA,BOSB^a^ ↑ Muscle glucose ↑Glycogen restore	(112)
2	Manchurian walnut	100 mice	Control: Deionized water Sample: Manchurian Walnut hydrolysate	Control: *Ad libitum* Sample 800 mg/kg BW/day 30 min before exercise 28 days	78.43%-(51 → 91 min)	↑Antioxidant enzymes ↓BUN, BLA,BOSB ↑ Muscle glucose ↑Glycogen restore Immunomodulatory	(113)
3	American Ginseng	240 mice	Control 1: Distilled water Control 2: Whey protein Sample: Gingseng peptides	Control 1:0.1mL/g BW/day Control 2: 450 mg/kg BW/day Sample225–900 mg/kg BW/day 30 days	Control 2 (15min) 35.24%- (14 → 19.93 min) 82.62%- (14 → 25.5min)	↑Antioxidant enzymes ↓BUN, BLA,BOSB ↑ Muscle glucose ↑Glycogen restore ↑ Mitochondrial function	(114)
4	Egg	30 mice	Control 1: Casein Control 2: Egg White protein Sample: Egg white hydrolysate	Controls and sample: 20% of its diet 14 days	90.9%- (2.2 → 4.2 min)	↑Antioxidant capacity	(115)
5	Pea	150 mice	Control 1: Distilled water Control 2: Pea protein isolate Sample: Pea peptides	Control 1: mL/g BW/day Control 2: 200 mg/kg BW/day Sample: 100–400 mg/kg BW/day30 days	Control 1: 22.3%- (10.41 → 12.73 min) 66.5%- (10.41 → 17.3 3 min) Control 2: 12.9%- (11.28 → 12.73 min) 53.6%- (11.28 → 17.33 min)	↑Antioxidant enzymes ↓BUN, BLA ↑ Muscle glucose ↑Glycogen restore Immunomodulatory Anti-inflammatory	(116)
6	Spirulina	80 mice	Control: Distilled water Sample: Spirulina peptides	Control: mL/g BW/day Samples:125–2,000 mg/kg BW/day 28 days	177.7%- (20 → 55.54 min) 264.9%- (20 → 72.98 min)	↑Antioxidant enzymes ↓ BLA ↑ Fat catabolism	(117)

a *Body weight (BW), Blood Urea Nitrogen (BUN) Blood Lactic Acid (BLA), Blood Oxidative stress biomarkers (BOSB)*.

b *Increase of time of exhaustion: In comparison with control group. In the case that two values appear, the first one coincides with the group that consumed the lowest dose of hydrolysate and the second one with the highest dose of hydrolysate*.

The mechanism of action of anti-fatigue peptides can be investigated by analyzing several biomarkers. Depletion of glycogen stores in muscle and liver results in a reduction of available blood glucose, leading to hypoglycaemia and fatigue (118). Therefore, maintenance of glycogen content may result in decreased fatigue generation. According to Table 2, anti-fatigue peptides could induce glycogen turnover through activation of glycogen synthase in skeletal muscle and improve blood insulin levels (116). Moreover, it has been postulated that some of these peptides upregulated the AMPK signaling pathway (117, 119), which could induce an increase of the fat catabolism (120). Glycerol is produced by lipolysis and could act positively as a direct substrate for gluconeogenesis, enhancing the regeneration of glycogen in liver and muscle (121). In certain situations, proteins can be used as energy source and urea is the main metabolite from protein catabolism and one of the main blood biomarkers of fatigue (122). This compound is produced in the liver, transported to the kidney and then excreted in the urine. The results of different assays showed that the peptides did not promote urea excretion, suggesting that preservation of glycogen storage could be the reason for the decrease in blood urea levels. Therefore, it suggests that the maintenance of glucose prevents the consumption of muscle protein as an energy substrate (116). The main metabolite of anaerobic catabolism of glucose is lactic acid. This compound in excess can lead to a reduction in muscular endurance through acidosis, that is one of the main causes of fatigue in the human body (123). According to some authors, referred to in Table 2, these peptides could also increase the lactate dehydrogenase activity, which catalyzes the formation of pyruvate from lactic acid, leading to a reduction in lactic acid, and thus a possible reduction in fatigue (112).

Other important factor related to fatigue is oxidative stress. In this situation, it seemed that anti-fatigue peptides could act as antioxidant agents. They can act by reducing blood oxidative stress biomarkers, such as malonaldehyde (MDA) and by improving the activity of antioxidant enzymes (113, 121), such as superoxide dismutase (SOD), which removes oxygen radicals in the organism, and glutathione peroxidase (GSH-Px), that contributes to the elimination of peroxides *in vivo* (124). The increase in SOD and GSH-Px activities could also reduce oxidative stress, cell membrane and muscle fibers damage by the reduction in ROS, allowing an increase of training time without exhaustion (113, 125). There is also a correlation between fatigue and the immune system. When the body feels depleted, the action of the immune system is often reduced, resulting in increased vulnerability to disease (126). In this context, some peptides could enhance immune response by increasing the concentration of phagocytes and IgA, which represent the first-line defense. These peptides also decreased the levels of proinflammatory cytokines IL-6 and TNF-α, which could help maintain normal immune system function, and could have a positive impact on reducing fatigue, because if there is less inflammation in muscle fibers, this body tissue could work longer without soreness (116, 127).

Although in most cases animal studies have been performed to analyse the effect on the reduction of physical fatigue, some human studies have also been carried out. Oe et al. (128), performed a randomized, double-blind, controlled trial, to evaluate the ability of peptides from an egg white hydrolysate (EWH) on mental fatigue in athletes. The results showed that the 2-week intake of 5 g/day of EWH 1 h before exercise, induced a significant reduction in mental fatigue compared to a placebo group (128). According to the authors, it seemed that the mechanism involved in this effect is associated with the promotion of nitric acid-related signaling (128, 129).

In summary, anti-fatigue peptides obtained from natural sources may be a very interesting supplement to increase the performance time of an athlete, being especially interesting in endurance sports. However, further studies in humans are needed to confirm this effect.

### Protein Hydrolysates in Sports Rehabilitation

An adequate and balanced diet is very important in recovery and rehabilitation. Specialized nutrition for recovery from injury in athletes follows similar dietary patterns to those of general sports nutrition, except for differences concerning the risk prevention of reduced anabolic sensitivity, sarcopenia, malnutrition or dysphagia, among others (130). Reduced anabolic sensitivity could be defined as the reduction of the MPS produced by several factors, such as aging, decreased in physical activity (131), reduced insulin sensibility or attenuation of aminoacidemia (132). If left untreated, this complication can lead to sarcopenia. Sarcopenia is a condition characterized by extreme muscular failure due to a reduction in the quality and quantity of skeletal muscle tissue (133). This disorder may also be related to inflammation (134), malnutrition related to loss of appetite (135) and oxidative stress (136). An increase in protein intake (137), and physical exercise (138) are the main recommendations to reduce the effects of this condition. Recently, it has been observed that protein sources such as fish (139) or whey (140) could give very promising results in the production of hydrolysates for the treatment of sarcopenia. In fact, protein hydrolysates could be considered a very beneficial supplement for subjects with this condition. On the one hand, their insulinotropic effect and their increase in aminoacidemia could facilitate, with a lower dose than intact proteins, the MPS of their muscles, despite anabolic resistance. On the other hand, the bioactive peptides generated after hydrolysis could exert an anti-inflammatory and antioxidant effect, reducing the risk generated by oxidative stress (141). Finally, another advantage of hydrolysates is that, being pre-digested, they may have a smooth texture that could be easier to swallow (142). This property could help protein intake in patients with dysphagia and mouth and throat problems, disorders closely related to aging (143).

In conclusion, hydrolysates have demonstrated several advantages over intact proteins, such as a higher speed in the MPS or the production of peptides with specific bioactivities that could act from a multifunctional point of view, in the improvement of sport performance and their related disorders.

## Amino Acids

In previous sections, it has been noted that the amino acids are responsible for several benefits attributed to proteins in sports nutrition. Among the amino acids, the most studied and the most consumed as supplements are BCAAs (leucine, valine and isoleucine), glutamine and β-alanine (144).

In recent decades, the study of the ergogenic effect of amino acids present in vegetable sources has increased, with citrulline and theanine being the most outstanding.

L-citrulline is a non-essential amino acid found mainly in watermelon (145). In the last decade, it has been observed that this amino acid could exert an ergogenic effect through two main pathways. L-citrulline is a potent endogenous precursor of L-arginine which acts as a precursor of nitric oxide (NO) (146). NO has numerous functions in the body, the most prominent being its vasodilator effect, which increases blood flow to the muscle, thereby increasing the bio accessibility of nutrients and the excretion of muscle waste products (147). On the other hand, citrulline is an intermediary metabolite of the urea cycle, which exerts an anti-fatigue effect by enhancing the excretion of ammonia produced by muscle catabolism during strenuous exercise (148). The reduction of ammonia levels in muscle could be reflected in a reduction of muscle glycolysis and lactate production (149). In recent studies, citrulline appears to show other ergogenic effect such as antioxidant capacity, immunomodulation, energy production and increase of MPS (150–152).

Theanine is an amino acid whose main source is green tea, and it is responsible for its characteristic flavor (153). This amino acid has demonstrated numerous beneficial properties, highlighting its high antioxidant and antihypertensive capacity (154). Regarding its ergogenic effect, it has been observed that theanine could improve movement accuracy (155) and possibly exerts an immunomodulatory and anti-inflammatory effect by contributing to the balance of th1/th2 leukocytes (156, 157). On the other hand, the demands of training and performance can be a source of stress and anxiety for elite athletes, and can negatively affect their health (158). Recently, it has been analyzed that theanine could exert a relaxing effect, reducing stress levels by blocking pro-stress glutamine signaling and increasing alpha waves in the brain (159). All this could indicate that theanine could exert a very interesting positive effect on stress reduction in athletes.

In summary, these alternative amino acids from plant sources could have a fundamental role in sports nutrition for their effect on many functions related to exercise.

## Emerging Strategies and Forthcoming Research

### Protein Blends

In the previous sections, it has been shown that each protein source contains a different set of beneficial properties. Therefore, it is possible to think that a blended protein supplement can potentially provide greater benefit by combining their ergogenic effects (160). Several studies have evaluated the effect of soy-dairy or vegetable-dairy protein blend supplementation in athletes. The hypothesis was that the ergogenic effect could be improved by combining the MPS-enhancing capacity of whey with the antioxidant and anti-inflammatory activity of soy and other plant-based proteins (161). The results showed that vegetable-dairy protein blends could exert ergogenic effects such as a reduction of fatigue (162), a slight enhancement of lean body mass (163) or an equal increase of MPS and more balanced post-prandial amino acid availability compared to whey (52, 164, 165). In general, vegetable proteins are deficient in certain essential amino acids, such as methionine in pulses, and cysteine and lysine in cereals (27). Protein blends could also be a promising solution to improve the quality of vegetable protein and favoring a greater ergogenic effect (166). In this regard, Brennan et al. (167), compared the effect of plant-based protein mixtures to whey protein. The results indicated that plant protein mixtures were not bioequivalent to whey protein, although they observed a faster increase of blood hyper aminoacidemia than individual vegetable proteins (167).

Thus, protein mixtures are an interesting line of research, as the combination of the ergogenic effects of different protein sources could be beneficial in sports supplementation.

### Probiotic and Protein

As we previously described, proteins have many benefits for athletes. However, a high protein intake together with exercise could induce oxidative stress, that could have a negative impact on the gut microbiota (13, 168). The intestinal microbiota contributes to the regulation of host physiological processes and plays a key role in the maintenance of intestinal homeostasis, nutrient absorption and the synthesis of bioactive metabolites such as EAA, short-chain fatty acids and vitamins (169). It has been observed that dietary proteins can exert a regulatory effect on the production of these beneficial metabolites, so an excess in protein intake can lead to adverse effects, generating a mismatch in the gut microbiota (170).

The negative effects of high-protein diets on the microbiota might be reduced by two strategies: the addition of prebiotics or probiotics. According to International Society of Sports Nutrition (ISSN), probiotics might also contribute positively to the improvement of athlete's health by increasing the integrity of the gut-barrier function, enhancing its immune system or reducing muscle damage (171). In this context, Jäger et al. (172), observed that the probiotic *Bacillus coagulans* GBI-30, 6,086 improved the absorption of proteins and amino acids (172). Therefore, they decided to evaluate its *in vitro* and *in vivo* synergistic effects in conjunction with plant-based proteins in athletes. In this trial, it was observed that the absorption rate of amino acids was higher than the absorption in the placebo group. It was considered that this effect could happen due to the proteolytic enzymes contained in the probiotics, which acted in the large intestine using undigested proteins and peptides as substrate, therefore generating more easily absorbable products (166). In a more recent study, the effect of this strain was tested with milk proteins, obtaining similar results (173).

Taken in total, the combination of probiotics with proteins could reduce the quality differences of protein sources naturally lower in leucine, such as some plant proteins, improving their bioactive properties, and possibly overcoming the microbiota problems related to high protein diet.

## Conclusion

In conclusion, protein supplementation in sports nutrition is a very broad field that includes proteins, hydrolysates, peptides, and amino acids. It encompasses very different areas as sport performance (increase energy, reduce fat body mass, maintain strength muscle, promote muscle protein synthesis, recovery muscular mass, or prevent nutritional deficiencies), sport-related complications (joint soreness, fatigue, sport injures and rehabilitation) and disciplines, such as food chemistry, immunology or physiology.

Although protein quality is generally lower in plants than in animals, there are other novel sources of protein, both animal and vegetable, whose effects on sports activity could be very interesting along with other environmental reasons to choose them as sports supplements. The future directions of these investigations highlight an interest on the development of protein products that not only improve sports nutrition, but also contain added value, such as a higher bioactive capacity or greater sustainability. In fact, the UN Nutrition aims to use resources more efficiently and reduce greenhouse gas emissions to secure the future of food and the environment. In fact, they suggested that a replacement of basic animal protein sources such as milk and eggs with more sustainable food alternatives is needed.

In this context, plant protein sources contain compounds that can help to protect the body against high levels of oxidative stress in exercise. Insect proteins are also a good-quality protein source, which can become a viable ingredient in liquid or solid food sports products, and mycoproteins could have more anabolic effect than traditional sport supplement protein with a much-reduced carbon footprint. Moreover, protein hydrolysates or their bioactive peptides could be especially beneficial from a multifunctional point of view, to improve sport performance, complications associated to the sport practice and healthy aging.

It is a fact that, in recent years, the demand for alternative proteins has been growing considerably and it is estimated that it will continue to do so in the next 20 years. In this context, sports nutrition will be an interesting target market and those new products that combine protein quality and bioactivity, including ergogenic capacity, and are friendly to the environment will be better accepted by athletes. Although alternative proteins present a great potential as natural source of biologically active compounds, the scientific evidence related to the biological properties of new sources of protein is almost limited to *in vitro* assays and it will be necessary more studies in animal models and human trials to evaluate their efficacy, safety, and their impact in human health before its commercialization.

## Author Contributions

ML-M and MM: conceptualization. ML-M: writing—original draft preparation. ML-M, MG-R, and MM: writing—review and editing. MG-R and MM: supervision. All authors contributed to the article and approved the submitted version.

## Funding

This work was supported by the Spanish Ministry of Education, Science and Universities (MICINN) (AGL2017-89213) and Francisco de Vitoria University (UFV 2019-09).

## Conflict of Interest

The authors declare that the research was conducted in the absence of any commercial or financial relationships that could be construed as a potential conflict of interest.

## Publisher's Note

All claims expressed in this article are solely those of the authors and do not necessarily represent those of their affiliated organizations, or those of the publisher, the editors and the reviewers. Any product that may be evaluated in this article, or claim that may be made by its manufacturer, is not guaranteed or endorsed by the publisher.

## References

[1] Vuori IM. Health benefits of physical activity with special reference to interaction with diet. Public Health Nutr. (2001) 4:517–28. 10.1079/PHN2001137 11683546

[2] Walker KZ O'Dea K Gomez M Girgis S Colagiuri R. Diet and exercise in the prevention of diabetes. J Hum Nutr Diet. (2010) 23:344–52. 10.1111/j.1365-277X.2010.01061.x 20337844

[3] Balagué N Torrents C Hristovski R Kelso JAS. Sport science integration: an evolutionary synthesis. Eur J Sport Sci. (2017) 17:51–62. 10.1080/17461391.2016.1198422 27685425

[4] Thomas DT Erdman KA Burke LM. American College of Sports Medicine Joint Position Statement. Nutrition and athletic performance. Med Sci Sports Exerc. (2016) 48:543–68. 10.1249/MSS.0000000000000852 26891166

[5] Maughan RJ. Nutritional ergogenic aids and exercise performance. Nutr Res Rev. (1999) 12:255–80. 10.1079/095442299108728956 19087454

[6] Tarnopolsky MA. Caffeine and creatine use in sport. Ann Nutr Metab. (2010) 57(Suppl. 2):1–8. 10.1159/000322696 21346331

[7] McNaughton LR Gough L Deb S Bentley D Sparks SA. Recent developments in the use of sodium bicarbonate as an ergogenic aid. Curr Sports Med Rep. (2016) 15:233–44. 10.1249/JSR.0000000000000283 27399820

[8] Jäger R Kerksick CM Campbell BI Cribb PJ Wells SD Skwiat TM. International society of sports nutrition position stand: protein and exercise. J Int Soc Sports Nutr. (2017) 14:20. 10.1186/s12970-017-0177-8 28642676 PMC5477153

[9] Kreider RB Kalman DS Antonio J Ziegenfuss TN Wildman R Collins R. International Society of Sports Nutrition position stand: safety and efficacy of creatine supplementation in exercise, sport, and medicine. J Int Soc Sports Nutr. (2017) 14:18. 10.1186/s12970-017-0173-z 28615996 PMC5469049

[10] Arenas-Jal M Suñé-Negre JM Pérez-Lozano P García-Montoya E. Trends in the food and sports nutrition industry: a review. Crit Rev Food Sci Nutr. (2020) 60:2405–21. 10.1080/10408398.2019.1643287 31352832

[11] Orrù S Imperlini E Nigro E Alfieri A Cevenini A Polito R. Role of functional beverages on sport performance and recovery. Nutrients. (2018) 10:1470. 10.3390/nu10101470 30308976 PMC6213308

[12] Watford M Wu G. Protein. Adv Nutr. (2018) 9:651–3. 10.1093/advances/nmy027 30060014 PMC6140426

[13] Jang L-G Choi G Kim S-W. Kim B-Y. Lee S Park H. The combination of sport and sport-specific diet is associated with characteristics of gut microbiota: an observational study. J Int Soc Sports Nutr. (2019) 16:21. 10.1186/s12970-019-0290-y 31053143 PMC6500072

[14] Ziegenfuss TN Landis JA Lemieux RA. Protein for sports-new data and new recommendations. Strength Cond J. (2010) 32:65–70. 10.1519/SSC.0b013e3181c16f83

[15] Bagheri R Hooshmand Moghadam B Jo E Tinsley GM Stratton MT Ashtary-Larky D. Comparison of whole egg v. egg white ingestion during 12 weeks of resistance training on skeletal muscle regulatory markers in resistance-trained men. Br J Nutr. (2020) 124:1035–43. 10.1017/S0007114520002238 32576297

[16] Harris RA Joshi M Jeoung NH. Mechanisms responsible for regulation of branched-chain amino acid catabolism. Biochem Biophys Res Commun. (2004) 313:391–6. 10.1016/j.bbrc.2003.11.007 14684174

[17] VanDusseldorp TA Escobar KA Johnson KE Stratton MT Moriarty T Cole N. Effect of branched-chain amino acid supplementation on recovery following acute eccentric exercise. Nutrients. (2018) 10:1389. 10.3390/nu10101389 30275356 PMC6212987

[18] Yu Y-M Fukagawa NK. Protein and amino acids. In: Marriott BP Birt DF Stallings VA Yates AA, editors. Present Knowledge in Nutrition. Volume Nutrition and Metabolism. 11th ed. London: Elsevier (2020). p. 15–35.

[19] Trommelen J Tomé D van Loon LJC. Gut amino acid absorption in humans: concepts and relevance for postprandial metabolism. Clin Nutr Open Sci. (2021) 36:43–55. 10.1016/j.nutos.2020.12.006

[20] Food and Agriculture Organization of the United Nations (FAO). Dietary Protein Quality Evaluation in Human Nutrition. (2013). paper 92. ISSN 0254-4725. Available online: http://www.fao.org/3/i3124e/i3124e.pdf (accessed at September 21, 2021).

[21] Wilson J Wilson GJ. Contemporary issues in protein requirements and consumption for resistance trained athletes. J Int Soc Sports Nutr. (2006) 3:7–27. 10.1186/1550-2783-3-1-7 18500966 PMC2129150

[22] Bulut Solak B Akin N. Health benefits of whey protein: a review. J Food Sci Eng. (2012) 2:129–37. 10.17265/2159-5828/2012.03.001

[23] Burd NA Yang Y Moore DR Tang JE Tarnopolsky MA Phillips SM. Greater stimulation of myofibrillar protein synthesis with ingestion of whey protein isolate v. micellar casein at rest and after resistance exercise in elderly men. Br J Nutr. (2012) 108:958–62. 10.1017/S0007114511006271 22289570

[24] Davies RW Carson BP Jakeman PM. The effect of whey protein supplementation on the temporal recovery of muscle function following resistance training: a systematic review and meta-analysis. Nutrients. (2018) 10:221. 10.3390/nu10020221 29462923 PMC5852797

[25] Taylor LW Wilborn C Roberts MD White A Dugan K. Eight weeks of pre- and postexercise whey protein supplementation increases lean body mass and improves performance in Division III collegiate female basketball players. Appl Physiol Nutr Metab. (2016) 41:249–54. 10.1139/apnm-2015-0463 26842665

[26] Patel S. Functional food relevance of whey protein: a review of recent findings and scopes ahead. J Funct Foods. (2015) 19:308–19. 10.1016/j.jff.2015.09.040

[27] Gorissen SHM Crombag JJR Senden JMG Waterval WAH Bierau J Verdijk LB. Protein content and amino acid composition of commercially available plant-based protein isolates. Amino Acids. (2018) 50:1685–95. 10.1007/s00726-018-2640-5 30167963 PMC6245118

[28] Chapman A. Numbers of Living Species in Australia and the World. 1st ed. (2006). Available online at: https://www.environment.gov.au/system/files/pages/2ee3f4a1-f130-465b-9c7a-79373680a067/files/nlsaw-2nd-complete.pdf (accessed July 21, 2021).

[29] Murefu TR Macheka L Musundire R Manditsera FA. Safety of wild harvested and reared edible insects: a review. Food Control. (2019) 101:209–24. 10.1016/j.foodcont.2019.03.003

[30] Zielinska E Baraniak B Karas M. Comparison of functional properties of edible insects and protein preparations thereof. Lebenson Wiss Technol. (2018) 91:168–74. 10.1016/j.lwt.2018.01.058

[31] Regulation (EU) 2015/228 of 17 February 2015 replacing Annexes I to VII to Council Regulation (EC) No 4/2009 on Jurisdiction, Applicable Law, Recognition and Enforcement of Decisions and Cooperation in Matters Relating to Maintenance Obligations. Available online at: https://eur-lex.europa.eu/legal-content/EN/ALL/?uri=CELEX%3A32013R0609 (accessed September 10, 2021).

[32] Van Huis A Van Itterbeeck J Klunder H Mertens E Halloran A Muir G. Edible Insects: Future Prospects for Food and Feed Security. Food and Agriculture Organization of the United Nations (2013). Available online at: http://www.fao.org/3/i3253e/i3253e.pdf (accessed July 21, 2021).

[33] Kim T-K Yong HI Kim Y-B Kim H-W Choi Y-S. Edible insects as a protein source: a review of public perception, processing technology, and research trends. Food Sci Anim Resour. (2019) 39: 521–40. 10.5851/kosfa.2019.e53 31508584 PMC6728817

[34] Churchward-Venne TA Pinckaers PJM van Loon JJA van Loon LJC. Consideration of insects as a source of dietary protein for human consumption. Nutr Rev. (2017) 75:1035–45. 10.1093/nutrit/nux057 29202184

[35] World Health Organization (WHO). Protein and Amino Acid Requirements in Human Nutrition. Singapore: World Health Organization Technical Report Series (2007). p. 1. 18330140

[36] Yi L Lakemond CMM Sagis LMC Eisner-Schadler V van Huis A van Boekel MAJS. Extraction and characterisation of protein fractions from five insect species. Food Chem. (2013) 141:3341–8. 10.1016/j.foodchem.2013.05.115 23993491

[37] Duan Y Li F Liu H Li Y Liu Y Kong X. Nutritional and regulatory roles of leucine in muscle growth and fat reduction. Front Biosci. (2015) 20:796–813. 10.2741/4338 25553480

[38] Yi L Van Boekel MAJS Boeren S Lakemond CMM. Protein identification and *in vitro* digestion of fractions from Tenebrio molitor. Eur Food Res Technol. (2016) 242:1285–97. 10.1007/s00217-015-2632-6

[39] Nongonierma AB FitzGerald RJ. Unlocking the biological potential of proteins from edible insects through enzymatic hydrolysis: a review. Innov Food Sci Emerg Technol. (2017) 43:239–52. 10.1016/j.ifset.2017.08.014

[40] Vangsoe MT Thogersen R Bertram HC Heckmann L-HL Hansen M. Ingestion of insect protein isolate enhances blood amino acid concentrations similar to soy protein in a human trial. Nutrients. (2018) 10:1357. 10.3390/nu10101357 30248987 PMC6212924

[41] Vangsoe MT Joergensen MS Heckmann L-HL Hansen M. Effects of insect protein supplementation during resistance training on changes in muscle mass and strength in young men. Nutrients. (2018) 10:335. 10.3390/nu10030335 29534456 PMC5872753

[42] Hermans WJH Senden JM Churchward-Venne TA Paulussen KJM Fuchs CJ Smeets JSJ. Insects are a viable protein source for human consumption: from insect protein digestion to postprandial muscle protein synthesis *in vivo* in humans: a double-blind randomized trial. Am J Clin Nutr. (2021) 114:934–44. 10.1093/ajcn/nqab115 34020450 PMC8408844

[43] Radnitz C Beezhold B DiMatteo J. Investigation of lifestyle choices of individuals following a vegan diet for health and ethical reasons. Appetite. (2015) 90:31–6. 10.1016/j.appet.2015.02.026 25725486

[44] Kuesten C Hu C. Functional foods and protein supplementation handbook of eating and drinking: interdisciplinary perspectives. In: Meiselman HL, editor. Handbook of Eating and Drinking: Interdisciplinary Perspectives. Cham: Springer International Publishing (2020). p. 941–64.

[45] Lynch HM Wharton CM Johnston CS. Cardiorespiratory fitness and peak torque differences between vegetarian and omnivore endurance athletes: a cross-sectional study. Nutrients. (2016) 8:726. 10.3390/nu8110726 27854281 PMC5133111

[46] Mangano KM Sahni S Kiel DP Tucker KL Dufour AB Hannan MT. Dietary protein is associated with musculoskeletal health independently of dietary pattern: the Framingham Third Generation Study. Am J Clin Nutr. (2017) 105:714–22. 10.3945/ajcn.116.136762 28179224 PMC5320406

[47] Shenoy S Dhawan M Singh Sandhu J. Four weeks of supplementation with isolated soy protein attenuates exercise-induced muscle damage and enhances muscle recovery in well trained athletes: a randomized trial. Asian J Sports Med. (2016) 7:e33528. 10.5812/asjsm.33528 27826398 PMC5098124

[48] Gorissen SH Horstman AM Franssen R Crombag JJ Langer H Bierau J. Ingestion of wheat protein increases *in vivo* muscle protein synthesis rates in healthy older men in a randomized trial. J Nutr. (2016) 146:1651–9. 10.3945/jn.116.231340 27440260

[49] Xia Z Cholewa JM Dardevet D Huang T Zhao Y Shang H. Effects of oat protein supplementation on skeletal muscle damage, inflammation and performance recovery following downhill running in untrained collegiate men. Food Funct. (2018) 9:4720–9. 10.1039/C8FO00786A 30094437

[50] Banaszek A Townsend JR Bender D Vantrease WC Marshall AC Johnson KD. The effects of whey vs. pea protein on physical adaptations following 8-weeks of high-intensity functional training (HIFT): a. pilot study Sports. (2019) 7:12. 10.3390/sports7010012 30621129 PMC6358922

[51] Lynch HM Buman MP Dickinson JM Ransdell LB Johnston CS Wharton CM. No significant differences in muscle growth and strength development when consuming soy and whey protein supplements matched for leucine following a 12 week resistance training program in men and women: a randomized trial. Int J Environ Res Public Health. (2020) 17:3871. 10.3390/ijerph17113871 32486007 PMC7312446

[52] Pinckaers PJM Kouw IWK Hendriks FK van Kranenburg JMX de Groot LCPGM Verdijk LB. No differences in muscle protein synthesis rates following ingestion of wheat protein, milk protein, and their protein blend in healthy, young males. Br J Nutr. (2021) 126:1–38. 10.1017/S0007114521000635 33597056

[53] Messina M Lynch H Dickinson JM Reed KE. No difference between the effects of supplementing with soy protein versus animal protein on gains in muscle mass and strength in response to resistance exercise. Int J Sport Nutr Exerc Metab. (2018) 28:674–85. 10.1123/ijsnem.2018-0071 29722584

[54] Bessada SM Barreira JC Oliveira MBP. Pulses and food security: Dietary protein, digestibility, bioactive and functional properties. Trends Food Sci Technol. (2019) 93:53–68. 10.1016/j.tifs.2019.08.022

[55] Reynaud Y Buffière C Cohade B Vauris M Liebermann K Hafnaoui N. True ileal amino acid digestibility and digestible indispensable amino acid scores (DIAASs) of plant-based protein foods. Food Chem. (2021) 338:128020. 10.1016/j.foodchem.2020.128020 32932087

[56] Samtiya M Aluko RE Dhewa T. Plant food anti-nutritional factors and their reduction strategies: An overview. Food Prod Process Nutr. (2020) 2:1–14. 10.1186/s43014-020-0020-5

[57] Omosebi MO Osundahunsi OF Fagbemi TN. Effect of extrusion on protein quality, antinutritional factors, and digestibility of complementary diet from quality protein maize and soybean protein concentrate. J Food Biochem. (2018) 42:e12508. 10.1111/jfbc.12508

[58] Hruby A Jacques PF. Dietary protein and changes in biomarkers of inflammation and oxidative stress in the Framingham Heart Study Offspring Cohort. Curr Dev Nutr. (2019) 3:nzz019. 10.1093/cdn/nzz019 31037277 PMC6483052

[59] Valko M Leibfritz D Moncol J Cronin MTD Mazur M Telser J. Free radicals and antioxidants in normal physiological functions and human disease. Int J Biochem Cell Biol. (2007) 39:44–84. 10.1016/j.biocel.2006.07.001 16978905

[60] Malaguti M Angeloni C Hrelia S. Polyphenols in exercise performance and prevention of exercise-induced muscle damage. Oxid Med Cell Longev. (2013) 2013:825928. 10.1155/2013/825928 23983900 PMC3742027

[61] Pingitore A Lima GPP Mastorci F Quinones A Iervasi G Vassalle C. Exercise and oxidative stress: potential effects of antioxidant dietary strategies in sports. Nutrition. (2015) 31:916–22. 10.1016/j.nut.2015.02.005 26059364

[62] Draganidis D Karagounis LG Athanailidis I Chatzinikolaou A Jamurtas AZ Fatouros IG. Inflammaging and skeletal muscle: can protein intake make a difference? J Nutr. (2016) 146:1940–52. 10.3945/jn.116.230912 27581584

[63] Higashida K Kim SH Higuchi M Holloszy JO Han D-H. Normal adaptations to exercise despite protection against oxidative stress. Am J Physiol Endocrinol Metab. (2011) 301:E779–84. 10.1152/ajpendo.00655.2010 21750271 PMC3214004

[64] HadŽović-DŽuvo A Valjevac A Lepara O Pjanić S HadŽimuratović A Mekić A. Oxidative stress status in elite athletes engaged in different sport disciplines. Bosn J basic Med Sci. (2014) 14:56–62. 10.17305/bjbms.2014.2262 24856375 PMC4333958

[65] Xiao CW. Health effects of soy protein and isoflavones in humans. J Nutr. (2008) 138:1244S−49S. 10.1093/jn/138.6.1244S 18492864

[66] Zhang T Zhao T Zhang Y Liu T Gagnon G Ebrahim J. Avenanthramide supplementation reduces eccentric exercise-induced inflammation in young men and women. J Int Soc Sports Nutr. (2020) 17:41. 10.1186/s12970-020-00368-3 32711519 PMC7382060

[67] Gaamouri N Zouhal H Hammami M Hackney AC Abderrahman A Ben Saeidi A. Ben Effects of polyphenol (carob) supplementation on body composition and aerobic capacity in taekwondo athletes. Physiol Behav. (2019) 205:22–8. 10.1016/j.physbeh.2019.03.003 30853622

[68] Tachibana N Yamashita Y Nagata M Wanezaki S Ashida H Horio F. Soy β-conglycinin improves glucose uptake in skeletal muscle and ameliorates hepatic insulin resistance in Goto-Kakizaki rats. Nutr Res. (2014) 34:160–7. 10.1016/j.nutres.2013.12.001 24461318

[69] Smulders MJM van de Wiel CCM van den Broeck HC van der Meer IM Israel-Hoevelaken TPM Timmer RD. Oats in healthy gluten-free and regular diets: a perspective. Food Res Int. (2018) 110:3–10. 10.1016/j.foodres.2017.11.031 30029703

[70] Finnigan TJA Wall BT Wilde PJ Stephens FB Taylor SL Freedman MR. Mycoprotein: the future of nutritious nonmeat protein, a symposium review. Curr Dev Nutr. (2019) 3:nzz021. 10.1093/cdn/nzz021 31187084 PMC6554455

[71] Finnigan T Needham L Abbott C, Mycoprotein: a healthy new protein with a low environmental impact. In: Nadathur SR Wanasundara JPD Scanlin L, editors. Sustainable Protein Sources. London: Elsevier (2017). p. 305–25.

[72] Coelho MOC Monteyne AJ Dunlop MV Harris HC Morrison DJ Stephens FB. Mycoprotein as a possible alternative source of dietary protein to support muscle and metabolic health. Nutr Rev. (2020) 78:486–97. 10.1093/nutrit/nuz077 31841152

[73] Hashempour-Baltork F Hosseini SM Assarehzadegan M-A Khosravi-Darani K Hosseini H. Safety assays and nutritional values of mycoprotein produced by Fusarium venenatum IR372C from date waste as substrate. J Sci Food Agric. (2020) 100:4433–41. 10.1002/jsfa.10483 32406520

[74] Hashempour-Baltork F Khosravi-Darani K Hosseini H Farshi P Reihani SFS. Mycoproteins as safe meat substitutes. J Clean Prod. (2020) 253:119958. 10.1016/j.jclepro.2020.119958

[75] Dunlop MV Kilroe SP Bowtell JL Finnigan TJA Salmon DL Wall BT. Mycoprotein represents a bioavailable and insulinotropic non-animal-derived dietary protein source: a dose-response study. Br J Nutr. (2017) 118:673–85. 10.1017/S0007114517002409 29017627

[76] Monteyne AJ Coelho MOC Porter C Abdelrahman DR Jameson TSO Jackman SR. Mycoprotein ingestion stimulates protein synthesis rates to a greater extent than milk protein in rested and exercised skeletal muscle of healthy young men: a randomized controlled trial. Am J Clin Nutr. (2020) 112:318–33. 10.1093/ajcn/nqaa092 32438401

[77] Monteyne AJ Coelho MOC Porter C Abdelrahman DR Jameson TSO Finnigan TJA. Branched-chain amino acid fortification does not restore muscle protein synthesis rates following ingestion of lower- compared with higher-dose mycoprotein. J Nutr. (2020) 150:2931–41. 10.1093/jn/nxaa251 32886108

[78] Monteyne AJ Dunlop MV Machin DJ Coelho MOC Pavis GF Porter C. A mycoprotein-based high-protein vegan diet supports equivalent daily myofibrillar protein synthesis rates compared with an isonitrogenous omnivorous diet in older adults: a randomised controlled trial. Br J Nutr. (2020) 3:1–11. 10.1017/S0007114520004481 33172506 PMC8110608

[79] Grimble GK. Mechanisms of peptide and amino acid transport and their regulation. Nestle Nutr Workshop Ser Clin Perform Programme. (2000) 3:63–8. 10.1159/000061797 11490614

[80] Manninen AH. Protein hydrolysates in sports nutrition. Nutr Metab. (2009) 6:38. 10.1186/1743-7075-6-38 19785737 PMC2761917

[81] Dale MJ Thomson RL Coates AM Howe PRC Brown A Buckley JD. Protein hydrolysates and recovery of muscle damage following eccentric exercise. Funct Foods Heal Dis. (2015) 5:34–43. 10.31989/ffhd.v5i1.164

[82] Moro T Brightwell CR Velarde B Fry CS Nakayama K Sanbongi C. Whey protein hydrolysate increases amino acid uptake, mTORC1 signaling, and protein synthesis in skeletal muscle of healthy young men in a randomized crossover trial. J Nutr. (2019) 149:1149–58. 10.1093/jn/nxz053 31095313 PMC7443767

[83] Yuan J Jiang B Li K Shen W Tang JL. Beneficial effects of protein hydrolysates in exercise and sports nutrition. J Biol Regul Homeost Agents. (2017) 31:183–8. 28337890

[84] Adibi SA Morse EL. Intestinal transport of dipeptides in man: relative importance of hydrolysis and intact absorption. J Clin Invest. (1971) 50:2266–75. 10.1172/JCI106724 5096512 PMC292168

[85] Sakata Y Yoshida C Fujiki Y Matsunaga Y Nakamura H Shimizu T. Effects of casein hydrolysate ingestion on thermoregulatory responses in healthy adults during exercise in heated conditions: a randomized crossover trial. Nutrients. (2020) 12:867. 10.3390/nu12030867 32213899 PMC7146450

[86] Morgan PT Breen L. The role of protein hydrolysates for exercise-induced skeletal muscle recovery and adaptation: a current perspective. Nutr Metab. (2021) 18:44. 10.1186/s12986-021-00574-z 33882976 PMC8061049

[87] Nongonierma AB O'Keeffe MB FitzGerald RJ. Milk protein hydrolysates and bioactive peptides BT - advanced dairy chemistry: volume 1B: proteins: applied aspects. In: McSweeney PLH O'Mahony JA, editors. Advanced Dairy Chemistry. New York, NY: Springer (2016). p. 417–82.

[88] Liu YF Oey I Bremer P Carne A Silcock P. Bioactive peptides derived from egg proteins: a review. Crit Rev Food Sci Nutr. (2018) 58:2508–30. 10.1080/10408398.2017.1329704 28609123

[89] Morifuji M Koga J Kawanaka K Higuchi M. Branched-chain amino acid-containing dipeptides, identified from whey protein hydrolysates, stimulate glucose uptake rate in L6 myotubes and isolated skeletal muscles. J Nutr Sci Vitaminol. (2009) 55:81–6. 10.3177/jnsv.55.81 19352067

[90] Iwasa M Takezoe S Kitaura N Sutani T Miyazaki H Aoi W. milk casein hydrolysate-derived peptide enhances glucose uptake through the AMP-activated protein kinase signalling pathway in skeletal muscle cells. Exp Physiol. (2021) 106:496–505. 10.1113/EP088770 33369793

[91] Muniyappa R Montagnani M Koh KK Quon MJ. Cardiovascular actions of insulin. Endocr Rev. (2007) 28:463–91. 10.1210/er.2007-0006 17525361

[92] Sasako T Ueki K. (Insulin/IGF-1 signaling and aging). Nihon Rinsho. (2016) 74:1435–40. 30557473

[93] Calbet JAL Holst JJ. Gastric emptying, gastric secretion and enterogastrone response after administration of milk proteins or their peptide hydrolysates in humans. Eur J Nutr. (2004) 43:127–39. 10.1007/s00394-004-0448-4 15168035

[94] Manninen AH. Hyperinsulinaemia, hyper aminoacidemia and post-exercise muscle anabolism: the search for the optimal recovery drink. Br J Sports Med. (2006) 40:900–5. 10.1136/bjsm.2006.030031 16950882 PMC2465040

[95] Maffulli N Longo UG Gougoulias N Caine D Denaro V. Sport injuries: a review of outcomes. Br Med Bull. (2011) 97:47–80. 10.1093/bmb/ldq026 20710023

[96] Felician FF Xia C Qi W Xu H. Collagen from marine biological sources and medical applications. Chem Biodivers. (2018) 15:e1700557. 10.1002/cbdv.201700557 29521032

[97] Oikawa SY Macinnis MJ Tripp TR McGlory C Baker SK Phillips SM. Lactalbumin, not collagen, augments muscle protein synthesis with aerobic exercise. Med Sci Sports Exerc. (2020) 52:1394–403. 10.1249/MSS.0000000000002253 31895298

[98] Oikawa SY McGlory C D'Souza LK Morgan AK Saddler NI Baker SK. randomized controlled trial of the impact of protein supplementation on leg lean mass and integrated muscle protein synthesis during inactivity and energy restriction in older persons. Am J Clin Nutr. (2018) 108:1060–8. 10.1093/ajcn/nqy193 30289425

[99] Fan J Zhuang Y Li B. Effects of collagen and collagen hydrolysate from jellyfish umbrella on histological and immunity changes of mice photoaging. Nutrients. (2013) 5:223–33. 10.3390/nu5010223 23344251 PMC3571645

[100] Clark KL Sebastianelli W Flechsenhar KR Aukermann DF Meza F Millard RL. 24-Week study on the use of collagen hydrolysate as a dietary supplement in athletes with activity-related joint pain. Curr Med Res Opin. (2008) 24:1485–96. 10.1185/030079908X291967 18416885

[101] Zdzieblik D Oesser S Gollhofer A König D. Improvement of activity-related knee joint discomfort following supplementation of specific collagen peptides. Appl Physiol Nutr Metab. (2017) 42:588–95. 10.1139/apnm-2016-0390 28177710

[102] Dressler P Gehring D Zdzieblik D Oesser S Gollhofer A König D. Improvement of functional ankle properties following supplementation with specific collagen peptides in athletes with chronic ankle instability. J Sports Sci Med. (2018) 17:298–304. 10.1016/j.jbmt.2018.09.037 29769831 PMC5950747

[103] Clifford T Ventress M Allerton DM Stansfield S Tang JCY Fraser WD. The effects of collagen peptides on muscle damage, inflammation and bone turnover following exercise: a randomized, controlled trial. Amino Acids. (2019) 51:691–704. 10.1007/s00726-019-02706-5 30783776

[104] Jendricke P Centner C Zdzieblik D Gollhofer A König D. Specific collagen peptides in combination with resistance training improve body composition and regional muscle strength in premenopausal women: a randomized controlled trial. Nutrients. (2019) 11:892. 10.3390/nu11040892 31010031 PMC6521629

[105] Praet SFE Purdam CR Welvaert M Vlahovich N Lovell G Burke LM. oral supplementation of specific collagen peptides combined with calf-strengthening exercises enhances function and reduces pain in achilles tendinopathy patients. Nutrients. (2019) 11:76. 10.3390/nu11010076 30609761 PMC6356409

[106] Prowting JL Bemben D Black CD Day EA Campbell JA. Effects of collagen peptides on recovery following eccentric exercise in resistance-trained males-a pilot study. Int J Sport Nutr Exerc Metab. (2020) 31:1–8. 10.1123/ijsnem.2020-0149 33186897

[107] Finsterer J. Biomarkers of peripheral muscle fatigue during exercise. BMC Musculoskelet Disord. (2012) 13:218. 10.1186/1471-2474-13-218 23136874 PMC3534479

[108] Kirmse M Oertzen-Hagemann V de Marées M Bloch W Platen P. Prolonged collagen peptide supplementation and resistance exercise training affects body composition in recreationally active men. Nutrients. (2019) 11:1154. 10.3390/nu11051154 31126103 PMC6566878

[109] Oertzen-Hagemann V Kirmse M Eggers B Pfeiffer K Marcus K de Marées M. Effects of 12 weeks of hypertrophy resistance exercise training combined with collagen peptide supplementation on the skeletal muscle proteome in recreationally active men. Nutrients. (2019) 11:1072. 10.3390/nu11051072 31091754 PMC6566884

[110] Boyas S Guével A. Neuromuscular fatigue in healthy muscle: underlying factors and adaptation mechanisms. Ann Phys Rehabil Med. (2011) 54:88–108. 10.1016/j.rehab.2011.01.001 21376692

[111] Twomey R Aboodarda SJ Kruger R Culos-Reed SN Temesi J Millet GY. Neuromuscular fatigue during exercise: methodological considerations, etiology and potential role in chronic fatigue. Neurophysiol Clin Neurophysiol. (2017) 47:95–110. 10.1016/j.neucli.2017.03.002 28434551

[112] Zhao Y-Q Zeng L Yang Z-S Huang F-F Ding G-F Wang B. Anti-fatigue effect by peptide fraction from protein hydrolysate of croceine croaker (*Pseudosciaena crocea*) swim bladder through inhibiting the oxidative reactions including DNA damage. Mar Drugs. (2016) 14:221. 10.3390/md14120221 27983570 PMC5192458

[113] Fang L Ren D Cui L Liu C Wang J Liu W. Antifatigue, antioxidant and immunoregulatory effects of peptides hydrolyzed from manchurian walnut (*Juglans mandshurica* Maxim) on mice grain oil. Sci Technol. (2018) 1:44–52. 10.3724/SP.J.1447.GOST.2018.18028

[114] Li D Ren J Zhang T Liu R Wu L Du Q. Anti-fatigue effects of small-molecule oligopeptides isolated from Panax quinquefolium L. in mice. Food Funct. (2018) 9:4266–73. 10.1039/C7FO01658A 30027191

[115] Matsuoka R Kimura M Uno S Shidara H Kunou M. Egg white hydrolysate improves fatigue due to short-term swimming load test in mice. Food Sci Nutr. (2018) 6:2314–20. 10.1002/fsn3.810 30510731 PMC6261203

[116] Feng T Huang Y Tang Z Wei D Mo J. Anti-fatigue effects of pea (*Pisum sativum* L) peptides prepared by compound protease. J Food Sci Technol. (2020) 58:2265–72. 10.1007/s13197-020-04737-3 33967323 PMC8076353

[117] Chen Y Wang F Zhou J Niu T Xuan R Chen H Wu W (2020). *In vivo* antifatigue activity of spirulina peptides achieved by their antioxidant activity and by acting on fat metabolism pathway in mice. Nat Prod Commun. (2020) 15:1934578X20946233. 10.1177/1934578X20946233

[118] Ørtenblad N Westerblad H Nielsen J. Muscle glycogen stores and fatigue. J Physiol. (2013) 591:4405–13. 10.1113/jphysiol.2013.251629 23652590 PMC3784189

[119] Nakagawasai O Yamada K Sakuma W Takahashi K Odaira T Yamagata R. novel dipeptide derived from porcine liver hydrolysate induces recovery from physical fatigue in a mouse model. J Funct Foods. (2021) 76:104312. 10.1016/j.jff.2020.104312

[120] Ke R Xu Q Li C Luo L Huang D. Mechanisms of AMPK in the maintenance of ATP balance during energy metabolism. Cell Biol Int. (2018) 42:384–92. 10.1002/cbin.10915 29205673

[121] Wang Y Kwon H Su X Wondisford FE. Glycerol not lactate is the major net carbon source for gluconeogenesis in mice during both short and prolonged fasting. Mol Metab. (2020) 31:36–44. 10.1016/j.molmet.2019.11.005 31918920 PMC6881678

[122] Jin H-M Wei P. Anti-fatigue properties of tartary buckwheat extracts in mice. Int J Mol Sci. (2011) 12:4770–80. 10.3390/ijms12084770 21954324 PMC3179131

[123] Hall MM Rajasekaran S Thomsen TW Peterson AR. Lactate: friend or foe. PM R. (2016) 8:S8–15. 10.1016/j.pmrj.2015.10.018 26972271

[124] Powers SK Lennon SL. Analysis of cellular responses to free radicals: focus on exercise and skeletal muscle. Proc Nutr Soc. (1999) 58:1025–33. 10.1017/S0029665199001342 10817171

[125] Chang YB Hong K-B Kim MG Suh HJ Jo K. Effect of the protein hydrolysate of rice syrup meal on the endurance exercise performance of BALB/c mice. Food Funct. (2021) 12:1338–48. 10.1039/D0FO02667K 33448266

[126] Mensah FKF Bansal AS Ford B Cambridge G. Chronic fatigue syndrome and the immune system: Where are we now? Neurophysiol Clin. (2017) 47:131–8. 10.1016/j.neucli.2017.02.002 28410877

[127] Yu Y Wu G Jiang Y Li B Feng C Ge Y. Sea cucumber peptides improved the mitochondrial capacity of mice: a potential mechanism to enhance gluconeogenesis and fat catabolism during exercise for improved antifatigue property. Oxid Med Cell Longev. (2020) 4604387. 10.1155/2020/4604387 32685094 PMC7335390

[128] Oe M Sakamoto H Nishiyama H Sasahara R Masuda Y Adachi M. Egg white hydrolyzate reduces mental fatigue: randomized, double-blind, controlled study. BMC Res Notes. (2020) 13:443. 10.1186/s13104-020-05288-8 32948224 PMC7501625

[129] Sawa T Zaki MH Okamoto T Akuta T Tokutomi Y Kim-Mitsuyama S. Protein S-guanylation by the biological signal 8-nitroguanosine 3',5'-cyclic monophosphate. Nat Chem Biol. (2007) 3:727–35. 10.1038/nchembio.2007.33 17906641

[130] Papadopoulou SK. Rehabilitation nutrition for injury recovery of athletes: the role of macronutrient intake. Nutrients. (2020) 12:2449. 10.3390/nu12082449 32824034 PMC7468744

[131] Breen L Stokes KA Churchward-Venne TA Moore DR Baker SK Smith K. Two weeks of reduced activity decreases leg lean mass and induces anabolic resistance of myofibrillar protein synthesis in healthy elderly. J Clin Endocrinol Metab. (2013) 98:2604–12. 10.1210/jc.2013-1502 23589526

[132] Dickinson JM Drummond MJ Coben JR Volpi E Rasmussen BB. Aging differentially affects human skeletal muscle amino acid transporter expression when essential amino acids are ingested after exercise. Clin Nutr. (2013) 32:273–80. 10.1016/j.clnu.2012.07.009 22889597 PMC3517689

[133] Cruz-Jentoft AJ Bahat G Bauer J Boirie Y Bruyère O Cederholm T. Sarcopenia: revised European consensus on definition and diagnosis. Age Ageing. (2019) 48:16–31. 10.1093/ageing/afz046 30312372 PMC6322506

[134] Dalle S Rossmeislova L Koppo K. The role of inflammation in age-related sarcopenia. Front Physiol. (2017) 8:1045. 10.3389/fphys.2017.01045 29311975 PMC5733049

[135] Landi F Calvani R Tosato M Martone AM Ortolani E Savera G. Anorexia of aging: risk factors, consequences, and potential treatments. Nutrients. (2016) 8:69. 10.3390/nu8020069 26828516 PMC4772033

[136] Nascimento CM Ingles M Salvador-Pascual A Cominetti MR Gomez-Cabrera MC Viña J. Sarcopenia, frailty and their prevention by exercise. Free Radic Biol Med. (2019) 132:42–9. 10.1016/j.freeradbiomed.2018.08.035 30176345

[137] Baum JI Kim I-Y Wolfe RR. Protein consumption and the elderly: what is the optimal level of intake? Nutrients. (2016) 8:359. 10.3390/nu8060359 27338461 PMC4924200

[138] Shad BJ Thompson JL Breen L. Does the muscle protein synthetic response to exercise and amino acid-based nutrition diminish with advancing age? A systematic review. Am J Physiol Metab. (2016) 311:E803–17. 10.1152/ajpendo.00213.2016 27555299

[139] Lees MJ Carson BP. The potential role of fish-derived protein hydrolysates on metabolic health, skeletal muscle mass and function in ageing. Nutrients. (2020) 12:2434. 10.3390/nu12082434 32823615 PMC7468851

[140] Gilmartin S O'Brien N Giblin L. Whey for sarcopenia; can whey peptides, hydrolysates or proteins play a beneficial role? Foods. (2020) 9:750. 10.3390/foods9060750 32517136 PMC7353484

[141] Vijaykrishnaraj M Prabhasankar P. Marine protein hydrolysates: their present and future perspectives in food chemistry – a review. RSC Adv. (2015) 5:34864–77. 10.1039/C4RA17205A

[142] Tavano OL. Protein hydrolysis using proteases: an important tool for food biotechnology. J Mol Catal B Enzym. (2013) 90:1–11. 10.1016/j.molcatb.2013.01.011

[143] Gallegos C Brito-de la Fuente E Clavé P Costa A Assegehegn G. Nutritional aspects of dysphagia management. Adv Food Nutr Res. (2017) 81:271–318. 10.1016/bs.afnr.2016.11.008 28317607

[144] Goron A Moinard C. Amino acids and sport: a true love story? Amino Acids. (2018) 50:969–80. 10.1007/s00726-018-2591-x 29855718

[145] Trexler ET Persky AM Ryan ED Schwartz TA Stoner L Smith-Ryan AE. Acute effects of citrulline supplementation on high-intensity strength and power performance: a systematic review and meta-analysis. Sports Med. (2019) 49:707–18. 10.1007/s40279-019-01091-z 30895562

[146] Suzuki T Morita M Kobayashi Y Kamimura A. Oral L-citrulline supplementation enhances cycling time trial performance in healthy trained men: double-blind randomized placebo-controlled 2-way crossover study. J Int Soc Sports Nutr. (2016) 13:6. 10.1186/s12970-016-0117-z 26900386 PMC4759860

[147] Joyner MJ Casey DP. Regulation of increased blood flow (hyperemia) to muscles during exercise: a hierarchy of competing physiological needs. Physiol Rev. (2015) 95:549–601. 10.1152/physrev.00035.2013 25834232 PMC4551211

[148] Breuillard C Cynober L Moinard C. Citrulline and nitrogen homeostasis: an overview. Amino Acids. (2015) 47:685–91. 10.1007/s00726-015-1932-2 25676932

[149] Wax B Kavazis AN Luckett W. Effects of supplemental citrulline-malate ingestion on blood lactate, cardiovascular dynamics, and resistance exercise performance in trained males. J Diet Suppl. (2016) 13:269–82. 10.3109/19390211.2015.1008615 25674699

[150] Jourdan M Nair KS Carter RE Schimke J Ford GC Marc J. Citrulline stimulates muscle protein synthesis in the post-absorptive state in healthy people fed a low-protein diet - A pilot study. Clin Nutr. (2015) 34:449–56. 10.1016/j.clnu.2014.04.019 24972455 PMC4309748

[151] Papadia C Osowska S Cynober L Forbes A. Citrulline in health and disease. Review on human studies. Clin Nutr. (2018) 37:1823–8. 10.1016/j.clnu.2017.10.009 29107336

[152] Gonzalez AM Trexler ET. Effects of citrulline supplementation on exercise performance in humans: a review of the current literature. J Strength Cond Res. (2020) 34:1480–95. 10.1519/JSC.0000000000003426 31977835

[153] Vuong QV Bowyer MC Roach PD. L-Theanine: properties, synthesis and isolation from tea. J Sci Food Agric. (2011) 91:1931–9. 10.1002/jsfa.4373 21735448

[154] Williams J Sergi D McKune AJ Georgousopoulou EN Mellor DD Naumovski N. The beneficial health effects of green tea amino acid l-theanine in animal models: promises and prospects for human trials. Phyther Res. (2019) 33:571–83. 10.1002/ptr.6277 30632212

[155] Zaragoza J Tinsley G Urbina S Villa K Santos E Juaneza A. Effects of acute caffeine, theanine and tyrosine supplementation on mental and physical performance in athletes. J Int Soc Sports Nutr. (2019) 16:56. 10.1186/s12970-019-0326-3 31771598 PMC6880365

[156] Williams J Kellett J Roach PD McKune A Mellor D Thomas J. l-Theanine as a functional food additive: its role in disease prevention and health promotion. Beverages. (2016) 2:13. 10.3390/beverages2020013

[157] Juszkiewicz A Glapa A Basta P Petriczko E Zołnowski K Machaliński B. The effect of L-theanine supplementation on the immune system of athletes exposed to strenuous physical exercise. J Int Soc Sports Nutr. (2019) 16:7. 10.1186/s12970-019-0274-y 30770758 PMC6377763

[158] Ford JL Ildefonso K Jones ML Arvinen-Barrow M. Sport-related anxiety: current insights. J Sport Med. (2017) 8:205–12. 10.2147/OAJSM.S125845 29138604 PMC5667788

[159] Williams JL Everett JM D'Cunha NM Sergi D Georgousopoulou EN Keegan RJ. The effects of green tea amino acid L-theanine consumption on the ability to manage stress and anxiety levels: a systematic review. Plant Foods Hum Nutr. (2020) 75:12–23. 10.1007/s11130-019-00771-5 31758301

[160] Reidy PT Walker DK Dickinson JM Gundermann DM Drummond MJ Timmerman KL. Protein blend ingestion following resistance exercise promotes human muscle protein synthesis. J Nutr. (2013) 143:410–6. 10.3945/jn.112.168021 23343671 PMC3738242

[161] Borack MS Reidy PT Husaini SH Markofski MM Deer RR Richison AB. Soy-dairy protein blend or whey protein isolate ingestion induces similar postexercise muscle mechanistic target of rapamycin complex 1 signaling and protein synthesis responses in older men. J Nutr. (2016) 146:2468–75. 10.3945/jn.116.231159 27798330 PMC5118761

[162] Ren G Yi S Zhang H Wang J. Ingestion of soy-whey blended protein augments sports performance and ameliorates exercise-induced fatigue in a rat exercise model. Food Funct. (2017) 8:670–9. 10.1039/C6FO01692H 28121323

[163] Reidy PT Borack MS Markofski MM Dickinson JM Deer RR Husaini SH. Protein supplementation has minimal effects on muscle adaptations during resistance exercise training in young men: a double-blind randomized clinical trial. J Nutr. (2016) 146:1660–9. 10.3945/jn.116.231803 27466602 PMC4997282

[164] Reidy PT Walker DK Dickinson JM Gundermann DM Drummond MJ Timmerman KL. Soy-dairy protein blend and whey protein ingestion after resistance exercise increases amino acid transport and transporter expression in human skeletal muscle. J Appl Physiol. (2014) 116:1353–64. 10.1152/japplphysiol.01093.2013 24699854 PMC4044402

[165] Liu J Klebach M Visser M Hofman Z. Amino acid availability of a dairy and vegetable protein blend compared to single casein, whey, soy, and pea proteins: a double-blind, cross-over trial. Nutrients. (2019) 11:2613. 10.3390/nu11112613 31683779 PMC6893549

[166] Jäger R Zaragoza J Purpura M Iametti S Marengo M Tinsley GM. Probiotic administration increases amino acid absorption from plant protein: a placebo-controlled, randomized, double-blind, multicenter, crossover study probiotics. Antimicrob Proteins. (2020) 12:1330–9. 10.1007/s12602-020-09656-5 32358640 PMC7641926

[167] Brennan JL Keerati-u-rai M Yin H Daoust J Nonnotte E Quinquis L. Differential responses of blood essential amino acid levels following ingestion of high-quality plant-based protein blends compared to whey protein—a double-blind randomized, cross-over. Clin Trial Nutr. (2019) 11:2987. 10.3390/nu11122987 31817691 PMC6950667

[168] Clark A Mach N. Exercise-induced stress behavior, gut-microbiota-brain axis and diet: a systematic review for athletes. J Int Soc Sports Nutr. (2016) 13:43. 10.1186/s12970-016-0155-6 27924137 PMC5121944

[169] Neis EPJG Dejong CHC Rensen SS. The role of microbial amino acid metabolism in host metabolism. Nutrients. (2015) 7:2930–46. 10.3390/nu7042930 25894657 PMC4425181

[170] Beaumont M Portune KJ Steuer N Lan A Cerrudo V Audebert M. Quantity and source of dietary protein influence metabolite production by gut microbiota and rectal mucosa gene expression: a randomized, parallel, double-blind trial in overweight humans. Am J Clin Nutr. (2017) 106:1005–19. 10.3945/ajcn.117.158816 28903954

[171] Jäger R Mohr AE Carpenter KC Kerksick CM Purpura M Moussa A. International society of sports nutrition position stand: probiotics. J Int Soc Sports Nutr. (2019) 16:62. 10.1186/s12970-019-0329-0 31864419 PMC6925426

[172] Jäger R Purpura M Farmer S Cash HA Keller D. Probiotic Bacillus coagulans GBI-30, 6086 improves protein absorption and utilization. Probiotics Antimicrob Proteins. (2018) 10:611–5. 10.1007/s12602-017-9354-y 29196920 PMC6208742

[173] Stecker RA Moon JM Russo TJ Ratliff KM Mumford PW Jäger R. Bacillus coagulans GBI-30, 6086 improves amino acid absorption from milk protein. Nutr Metab. (2020) 17:93. 10.1186/s12986-020-00515-2 33110439 PMC7585191

